# PARGT: a software tool for predicting antimicrobial resistance in bacteria

**DOI:** 10.1038/s41598-020-67949-9

**Published:** 2020-07-03

**Authors:** Abu Sayed Chowdhury, Douglas R. Call, Shira L. Broschat

**Affiliations:** 1 School of Electrical Engineering and Computer Science, Washington State University, P.O. Box 642752, Pullman, Washington USA; 2 Paul G. Allen School for Global Animal Health, Washington State University, P.O. Box 647090, Pullman, Washington USA; 3 Department of Veterinary Microbiology and Pathology, Washington State University, P.O. Box 647040, Pullman, Washington USA

**Keywords:** Computational models, Machine learning, Software, Computational science, Computer science, Software, Antimicrobials, Applied microbiology, Bacteria, Bioinformatics, Software

## Abstract

With the ever-increasing availability of whole-genome sequences, machine-learning approaches can be used as an alternative to traditional alignment-based methods for identifying new antimicrobial-resistance genes. Such approaches are especially helpful when pathogens cannot be cultured in the lab. In previous work, we proposed a game-theory-based feature evaluation algorithm. When using the protein characteristics identified by this algorithm, called ‘features’ in machine learning, our model accurately identified antimicrobial resistance (AMR) genes in Gram-negative bacteria. Here we extend our study to Gram-positive bacteria showing that coupling game-theory-identified features with machine learning achieved classification accuracies between 87% and 90% for genes encoding resistance to the antibiotics *bacitracin* and *vancomycin*. Importantly, we present a standalone software tool that implements the game-theory algorithm and machine-learning model used in these studies.

## Introduction

Antimicrobial resistance (AMR) refers to a property of bacteria when they become less susceptible to an antimicrobial agent^1–4^. Bacteria can gain AMR by overexpressing or duplicating available genes, undergoing chromosomal mutation, or obtaining resistance genes from other bacteria by means of horizontal gene transfer^1, 5^. According to a recently released report by the Centers for Disease Control and Prevention (CDC), at least 2.8 million people in the United States are infected every year by antimicrobial-resistant organisms, and these infections result in more than 35,000 deaths^6^. Also, according to a recently released report by the Organisation for Economic Co-operation and Development (OECD), 2.4 million deaths are predicted in Europe, North America, and Australia in the next 30 years due to antimicrobial-resistant infections, and such infections could cause up to US$3.5 billion in additional health care costs per year^7, 8^. As AMR becomes a threat worldwide, both economically and to public health^9–13^, there is an urgent need to develop a preclinical tool for efficient prediction of AMR.

One conventional strategy for identifying genetically-encoded mechanisms for AMR involves sequence assembly^14–17^ and read-based techniques^18–20^ that map sequence data directly to reference databases. Although these methods perform well for known and highly conserved AMR genes, they may produce an unacceptable number of false positives (genes predicted to encode resistance when they do not) for highly dissimilar sequences as was demonstrated previously for Gram-negative bacteria^21^. Machine-learning techniques can be applied as an alternative solution for predicting putative AMR genes. Rather than using sequence similarity, a machine-learning model detects features, i.e., characteristics of a protein sequence, that are unique to AMR genes. Several machine-learning methods have been proposed to identify novel AMR genes from metagenomic and pan-genome data^12, 22, 23^, but these methods used a small number of genetic features for predictions. Moreover, these approaches did not use a feature-selection strategy to remove irrelevant and redundant features that might compromise the accuracy of a machine-learning model.

We recently introduced a game-theory-based feature selection approach (“game theoretic dynamic weighting based feature evaluation”, or GTDWFE) predicated on the supposition that a single feature might provide limited predictive value, but that it might contribute to form a strong coalition when used with other features^21^. We applied our feature selection approach in Gram-negative bacteria and obtained prediction accuracies ranging from 93% to 99% for prediction of genes that encode resistance to acetyltransferase (*aac*), $\beta$-lactamase (*bla*), and dihydrofolate reductase (*dfr*). In our current study, we test the GTDWFE algorithm with data from Gram-positive bacteria. We then combine the results for both studies and introduce “Prediction of Antimicrobial Resistance via Game Theory” (PARGT), a software program with a graphical-user interface (GUI) that is designed to identify antimicrobial-resistance genes for both Gram-positive and -negative bacteria.

A major objective was to develop a software tool with a simple and intuitive GUI that is capable of extracting protein features without the need for manual curation and then use these features to identify putative AMR genes. PARGT integrates all of the tools and scripts required to identify protein features and to automatically generate feature subsets obtained via the GTDWFE algorithm. PARGT can be used with the Windows, Linux, or macOS, operating systems, and it provides options for predicting *bac* and *van* resistance genes in any Gram-positive bacteria and *aac*, *bla*, and *dfr* resistance genes in any Gram-negative bacteria. Users can test a single sequence or an entire genome for these genes. In addition, PARGT allows users to add newly confirmed AMR or non-AMR sequences to the training set as well as to reset the training data back to the original training set downloaded with the tool.

## Results

### Validation of PARGT

We validated the GTDWFE algorithm for feature selection as implemented previously^21^. In our earlier work, we considered the AMR (positive) and non-AMR (negative) amino-acid sequences of *aac*, *bla*, and *dfr* for *Acinetobacter*, *Klebsiella*, *Campylobacter*, *Salmonella*, and *Escherichia* as training datasets and tested our trained support vector machine (SVM)^24, 25^ machine-learning model with sequences from *Pseudomonas*, *Vibrio*, and *Enterobacter*. The combination of GTDWFE and SVM resulted in correct classification rates of 93%, 99%, and 97% for *aac*, *bla*, and *dfr*, respectively. This demonstrated that our approach was promising and that the GTDWFE algorithm is capable of identifying the most relevant, non-redundant, and interdependent features necessary for accurate prediction.

In this paper we consider validation of our GTDWFE model for AMR proteins in Gram-positive bacteria. We use the unique AMR and non-AMR sequences available for *bac* and *van* from the Gram-positive bacteria *Clostridium *spp. and *Enterococcus *spp. as the training datasets for our SVM model. These training datasets are used to generate the best feature subsets by means of the GTDWFE approach. The training datasets contain 25 and 52 AMR (positive) examples for *bac* and *van*, respectively. A total of 52 non-AMR examples are considered as negative samples for each of the training datasets. In the GTDWFE approach, we select features based on the relevance, non-redundancy, and interdependency values of all features. For this analysis, we need to set an interdependent group size $\delta$ to measure the interdependency between features, where $\delta$ is used in the computation of the Banzhaf power index^26^ and indicates the size of each feature group. We selected a value of $\delta =3$ based on previous work^21^ where we found that an interdependent group size of 3 was sufficient to identify best feature subsets from training datasets. We then test our trained model with known AMR and non-AMR samples from *Staphylococcus*, *Streptococcus*, and *Listeria*. The test datasets contain 6 and 9 AMR (positive) sequences for *bac* and *van*, respectively, and 14 non-AMR (negative) sequences are used for each test dataset.

**Table 1:** Predicted *bac* AMR sequences for *Staphylococcus*, *Streptococcus*, and *Listeria* using the GTDWFE algorithm.

NCBI accession number	Protein names	Note
AAF81096	Putative undecaprenol kinase	True positive
AAO04051	Undecaprenol kinase	True positive
BAE05519	bacA	True positive
BAE19180	Putative undecaprenol kinase bacitracin resistance protein	True positive
CAL27243	Putative undecaprenol kinase	True positive
EEK11594	Undecaprenyl-diphosphatase UppP	True positive
EUJ19660	Hypothetical protein MAQA_05683	False positive
WP_018370157	Serine *O*-acetyltransferase	False positive

**Table 2:** Predicted *van* AMR sequences for *Staphylococcus*, *Streptococcus*, and *Listeria* using the GTDWFE algorithm.

NCBI accession number	Protein names	Note
AAQ17160	Vancomycin/teicoplanin A-type resistance protein VanA (plasmid)	True positive
AAQ17159	Vancomycin resistance protein VanH (plasmid)	True positive
AAQ17157	Vancomycin response regulator VanR (plasmid)	True positive
AAQ17158	Sensor histidine kinase VanS (plasmid)	True positive
AAQ17161	Vancomycin B-type resistance protein VanX (plasmid)	True positive
AAL07292	D,D-dipeptidase VanXb, partial	True positive
AAQ17162	D-alanyl-D-alanine carboxypeptidase VanY (plasmid)	True positive
AAQ17163	vanZ protein (plasmid)	True positive
CDC71755	Putative uncharacterized protein	False positive
WP_018370157	Serine *O*-acetyltransferase	False positive

Tables 1 and 2 list the predicted *bac* and *van* AMR sequences from our test datasets, respectively. In each table, we provide the NCBI accession number^27^ for each protein sequence together with its name, and we note whether an AMR protein was correctly classified as AMR (true positive) or a non-AMR sequence was incorrectly classified as AMR (false positive). The GTDWFE algorithm successfully identified all six *bac* AMR genes (true positives). However, it missclassified 2 of the 14 non-AMR sequences as AMR (false positives). Therefore, the number of true positives, true negatives (negatives accurately classified), false positives, and false negatives (positives classified as negatives) for *bac* are 6, 12, 2, and 0, respectively, and the sensitivity, specificity, and accuracy for *bac* are 100%, 86%, and 90%, respectively. As shown in Table 2 for *van*, 8 of 9 AMR sequences were correctly classified as AMR (true positives) whereas 2 of 14 non-AMR sequences were classified as AMR (false positives). Therefore, the number of true positives, true negatives, false positives, and false negatives for *van* are 8, 12, 2, and 1, respectively, and the sensitivity, specificity, and accuracy for *van* are 89%, 86%, and 87%, respectively. Note that the two tables contain one hypothetical protein and one putative uncharacterized protein. We have categorized these two proteins as false positives because they were identified as essential (non-AMR) genes in the Pathosystems Resource Integration Center (PATRIC)^28, 29^. However, it is quite possible that PARGT correctly identified them as AMR proteins given the number of annotation errors in public databases^30^. CDC71755 is from a *Staphylococcus* organism identified from a metagenome sequence, and EUJ19660 is from a *Listeria aquatica* organism obtained from an environmental water sample.

### Performance comparison with BLASTp and Kalign tools

We also compared the performance of our GTDWFE algorithm with BLASTp (https://blast.ncbi.nlm.nih.gov/Blast.cgi?PAGE=Proteins) and Kalign^31^ (https://www.ebi.ac.uk/Tools/msa/kalign/) results using default parameter settings. The outcomes shown in Supplementary Table S1 are the percent identities for *bac* AMR and non-AMR samples from *Staphylococcus*, *Streptococcus*, and *Listeria* with respect to the *bac* AMR samples of *Clostridium* and *Enterococcus*. A percent identity for BLASTp and Kalign as low as 38.13% and 46.19%, respectively, are needed to identify all the *bac* AMR sequences; however, these low percent identities lead to 6 and 3 of 14 false positives for BLASTp and Kalign, respectively, in which non-AMR sequences are miscategorized. Therefore, the low percent identities for BLASTp and Kalign required to identify all AMR sequences increase the number of false positives for a set of diverse AMR sequences. In Supplementary Table S2 we show that the performances of BLASTp and Kalign when identifying *van* AMR sequences are actually better than that of the GTDWFE algorithm. This is due to the very high similarity (>98.5% identity) between the training AMR and test AMR datasets for *van*. When training and test sets share high similarity, BLASTp and Kalign are guaranteed to give good results. However, as in the case of *bac* for which the training and test AMR data similarity ranges between 38.13% and 41.01%, BLASTp does not perform well. For Kalign, the similarity ranges between 46.19% and 49.17% so that it performs better than BLASTp. However, the GTDWFE algorithm will outperform both BLASTp and Kalign because it does not use sequence similarity but rather protein features for prediction. BLASTp and Kalign do not predict; they match sequence similarity.

## Discussion

In this work, we implemented a software package PARGT and extended our earlier work of identifying AMR genes in Gram-negative to Gram-positive bacteria. PARGT integrates the required software tools and scripts needed to generate all protein features automatically, and it performs predictions on user-inputted sequences. Moreover, users can update PARGT by including their own known AMR and non-AMR sequences to train the machine-learning model to potentially improve prediction accuracy. As our previous work described the experimental results for Gram-negative bacteria, in this paper we only included prediction results for Gram-positive bacteria. Simulation results showed that PARGT can predict AMR sequences for Gram-positive bacteria with accuracy ranging from 87% to 90%. PARGT gave better results for *bac* due to the diversity of sequences available, but BLASTp and Kalign exhibited better performance in the case of *van* because of the high similarity of sequences. To generate evolutionary and secondary structure features, we used the Uniprot database (containing 538,585 FASTA sequences) as our reference database for relatively fast execution; however, more accurate values for these features can be obtained using large-scale protein databases such as UniRef90 or UniRef100 (http://www.uniprot.org/help/uniref) as target/reference databases. Note, however, that there is a trade-off between accuracy and computational time when using a large-scale reference database to generate features. A parallel version of PARGT would reduce the execution time of the tool for and ameliorate the use of large-scale reference databases.

## Methods

### GTDWFE algorithm for feature selection

Feature collection, feature extraction, calculation of feature values, and feature selection using the GTDWFE algorithm are explained in detail in previous works^21, 32^. Briefly, a total of 621D candidate features were collected by means of a thorough literature search, where D stands for dimension (some features are single values, i.e., 1D, while others are vector values, e.g., 20D for the 20 different amino acids). We extracted all 621D features from both our positive (AMR) and negative (non-AMR) datasets and calculated their values. The GTDWFE algorithm was then used to select features for use in our machine-learning model. The GTDWFE selects the best feature at each iteration based on the relevance, non-redundancy, and interdependency values of all features. Initially, the weights of all features are the same i.e., 1. The relevance of a feature to the target class (AMR or non-AMR) and the distance of the feature to other features are calculated using Pearson’s correlation coefficient and the Tanimoto coefficient, respectively. These calculations are performed for all features, and the feature with the highest summation of relevance and distance is chosen as the initial selected feature. The Banzhaf power index^26^ is then calculated to estimate the interdependency between the selected feature and the remaining features. We measure the contribution of each feature when it forms a group with other features, and the conditional mutual information is calculated to find the Banzhaf power index of the features. The weight of each remaining feature is updated by adding the product of the current weight and the Banzhaf power index to the feature selected previously. In other words, at each step, we readjust the weight of the remaining features dynamically based on the features selected in earlier steps. Thus, the weight of a candidate feature actually corresponds to the interdependence values with the earlier selected features. The feature with the highest summation of relevance and distance values multiplied by the revised weight is chosen as the next selected feature. This process is repeated until the desired number of features has been reached.

### Machine-learning algorithm

After identifying the best feature subset for use with our classifier by means of the GTDWFE algorithm, we trained an SVM machine-learning model using this feature subset. This binary classifier was then used for prediction. As was true for our previous work, in PARGT we tuned the SVM using the training datasets and chose the best SVM model to predict the AMR proteins in the test sequences. We considered 10-fold cross validation to tune the SVM model. The SVM model with a radial basis function (RBF) kernel and a *cost* value of 4 was identified as the best model for both *bac* and *van* training datasets. For the SVM, the RBF is used as a function in the kernel trick to implicitly transform the original space of the data to a high-dimensional space to make the data samples linearly separable, and the *cost* parameter is used to regulate the classification error.

**Figure 1:**
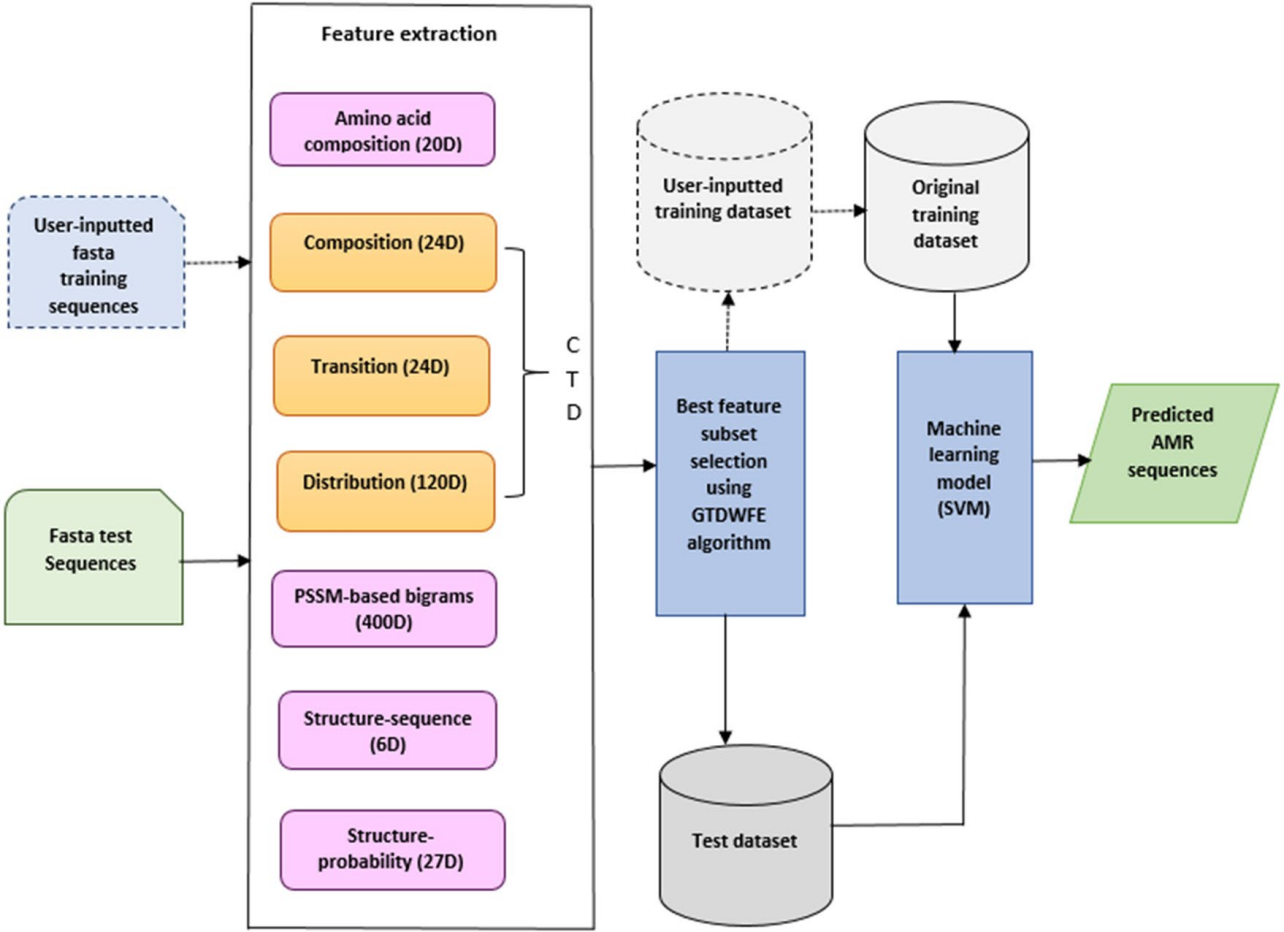
The components of PARGT. Components outlined by dotted lines indicate additional training samples supplied by a user.

### Overview of PARGT software

PARGT is an open-source software package designed and implemented for predicting antimicrobial resistance genes in bacteria. PARGT is written using both Python 3 and R. R scripts were written to identify physicochemical and secondary structure features and for machine-learning modeling, and Python 3 was used to run the R scripts, to generate position-specific scoring matrix (PSSM) features, and to implement the GUI. PARGT weight the importance of protein features based on their contributions during classification. All the required bioinformatics tools^33–39^ and scripts necessary to generate the protein features required in our machine-learning model are included in PARGT. PARGT uses the best feature subset identified by our GTDWFE algorithm to make predictions. It allows users to add new AMR and non-AMR sequences to the training datasets, and the software automatically updates the machine-learning model with the additional sequences, potentially resulting in an increase in the accuracy of the model. To minimize execution time, PARGT uses the UniProt database containing 538,585 protein sequences as a reference database, rather than a larger database, for generating PSSM and secondary structure features.

### Architecture of PARGT

**Figure 2:**
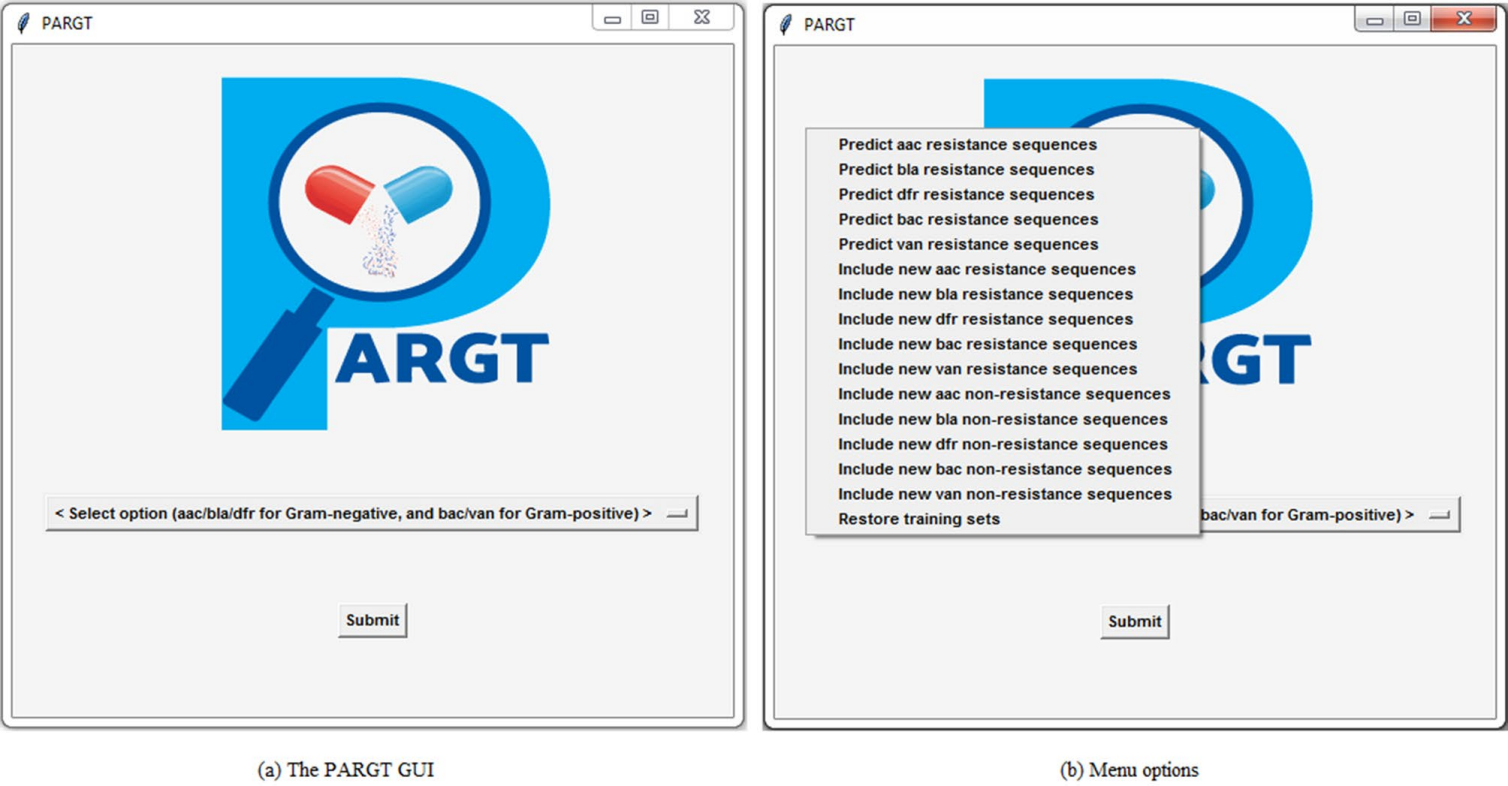
Illustration of the PARGT GUI with its pop-up menu.

Figures 1 and 2 depict the architecture and GUI for PARGT, respectively. PARGT allows a user to input a set of known AMR and non-AMR sequences to use in the training dataset, generating all required feature values for these sequences automatically. As shown in Fig. 1, the 20D amino acid composition feature vector, 168D feature vector based on the composition, transition and distribution (CTD) model^40, 41^, 400D feature vector based on the PSSM, and 33D feature vector based on the secondary structure sequence and secondary structure probability matrix are generated from the input protein sequences. Then the best feature subset is constructed using our GTDWFE feature selection algorithm. An SVM is used as the machine-learning model that is trained using the selected feature set. Recall that the SVM model used for PARGT is automatically tuned during the training phase. Finally, the trained SVM model is applied to predict AMR sequences from the test dataset.

As shown in Fig. 2, PARGT provides the option of predicting *aac*, *bla*, and *dfr* resistance genes for Gram-negative bacteria and *bac* and *van* resistance genes for Gram-positive bacteria. A user must select the appropriate option for predicting AMR from the GUI menu and also supply the test file for the set of protein sequences in FASTA format that they wish to have classified as AMR or non-AMR. PARGT automatically computes all the required feature values for the test sequences, and it provides an output file containing the set of predicted AMR sequences for the user’s test file. If a user wants to include new known AMR or non-AMR sequences to augment the training datasets, PARGT provides an option to do so for the five above-mentioned resistance classes. In addition, it provides the option of restoring the original training datasets in case a user decides they prefer to use them or or else wants to compare predictions using two different sets of training data.

### Datasets

We retrieved protein sequences for AMR genes from the Antibiotic Resistance Genes Database (ARDB)^42^, and non-AMR sequences were obtained from the PATRIC^28, 29^. Initially, we gathered 124 *bac* and 374 *van* AMR sequences for the Gram-positive bacteria *Clostridium* spp. and *Enterococcus* spp., and we randomly chose 52 essential protein sequences to use as non-AMR sequences. As many of the protein sequences were duplicates, CD-HIT^43, 44^ was applied to find unique sequences. A sequence identity of $\ge$ 90% was used as a threshold for removing duplicate sequences. After eliminating redundant protein sequences, our final counts were 25 *bac* and 52 *van* AMR sequences; none of the 52 non-AMR sequences were duplicates. We used this dataset to train our machine-learning model. In addition to the training dataset, we also gathered 102 *bac* and 22 *van* AMR sequences and 14 non-AMR sequences for the Gram-positive bacteria *Staphylococcus* spp., *Streptococcus* spp., and *Listeria* spp. from the data sources indicated above. We again applied CD-HIT to this dataset, and after the removal of duplicate sequences, 6 *bac* and 9 *van* AMR sequences and 14 non-AMR sequences remained. We used these as our test dataset to measure the accuracy of the classifier. The sequence identity of protein sequences could be as low as 10%. After validating our GTDWFE algorithm with the training and test sequences for the *bac* and *van* AMR classes, we again trained our classifier, but we used the sequences from all five bacterial genera, i.e., both training and test sequences, to potentially increase the accuracy of PARGT. The same retraining was also performed for our Gram-negative bacteria.

## Supplementary information

Supplementary Tables

## Data Availability

NCBI^27^ accession numbers for all proteins used in this work are listed in Supplementary Tables S3–S5. All experimental data are available at https://github.com/abu034004/PARGT.

## Code availability

The open source PARGT software package implemented for this work and the user’s manual are available at https://github.com/abu034004/PARGT.
