# Supplementary material for: PARGT: a software tool for predicting antimicrobial resistance in bacteria
Source: Sci Rep. 2020 Jul 3;10:11033. doi: 10.1038/s41598-020-67949-9 (PMC7335159; doi:10.1038/s41598-020-67949-9)
Supplement: Supplementary file 1 — Supplementary Tables [file 41598_2020_67949_MOESM1_ESM.pdf]

PARGT: A Software Tool for Predicting Antimicrobial Resistance in Bacteria

Abu Sayed Chowdhury[1,*], Douglas R. Call[1,2,3], and Shira L. Broschat[1,2,3]

[1]School of Electrical Engineering and Computer Science, Washington State University, P.O. Box 642752, Pullman, Washington, USA

[2]Paul G. Allen School for Global Animal Health, Washington State University, P.O. Box 647090, Pullman, Washington, USA

[3]Department of Veterinary Microbiology and Pathology, Washington State University, P.O. Box 647040, Pullman, Washington, USA

BLASTp and Kalign results for *Staphylococcus*, *Streptococcus*, and *Listeria*

Table S1: Percent identities for *bac* AMR and non-AMR samples from *Staphylococcus*, *Streptococcus*, and *Listeria* with respect to *bac* AMR sequences from *Clostridium* and *Enterococcus*.

**NCBI accession number**	**Protein name**	**BLASTp (% identity)**	**Kalign (% identity)**	**AMR (yes/no)**
AAF81096	putative undecaprenol kinase	38.83	46.19	Yes
AAO04051	undecaprenol kinase	40.15	48.94	Yes
BAE05519	bacA	38.62	47.64	Yes
BAE19180	putative undecaprenol kinase bacitracin resistance protein	41.01	49.17	Yes
CAL27243	putative undecaprenol kinase	38.13	46.84	Yes
EEK11594	undecaprenyl-diphosphatase UppP	38.64	46.78	Yes
EUJ19660	hypothetical protein MAQA_05683	37.04	34.41	No
EUJ18463	2-C-methyl-D-erythritol 2,4-cyclodiphosphate synthase	25.81	48.48	No
EUJ21751	lysyl-tRNA ligase	58.33	32.85	No
EUJ18464	glutamyl-tRNA ligase	0	35.61	No
EUJ17220	30S ribosomal protein S21	37.50	55	No
CDC69830	purine-nucleoside phosphorylase	0	35.56	No
CDC71755	putative uncharacterized protein	38.89	39.83	No
CDC68822	dNA gyrase subunit A	60.00	33.33	No
CDC68565	primosome assembly protein PriA	53.85	35.37	No
WP_018370009	HU family DNA-binding protein	40.91	48.72	No
WP_018370157	serine O-acetyltransferase	43.75	32.39	No
WP_018369970	DNA polymerase III subunit delta′	35.29	31.68	No
WP_018369925	segregation/condensation protein B	0	36.26	No
WP_018370631	chromosome segregation protein SMC	36.84	37.23	No

Table S2: Percent identities for *van* AMR and non-AMR samples of *Staphylococcus*, *Streptococcus*, and *Listeria* with respect to the *van* AMR sequences of *Clostridium* and *Enterococcus*.

**NCBI accession number**	**Protein name**	**BLASTp (% identity)**	**Kalign (% identity)**	**AMR (yes/no)**
AAQ17160	vancomycin/teicoplanin A-type resistance protein VanA (plasmid)	100	100	Yes
CAA94438	D-alanine:D-alanine ligase-related protein, partial	98.59	99.53	Yes
AAQ17159	vancomycin resistance protein VanH (plasmid)	100	100	Yes
AAQ17157	vancomycin response regulator VanR (plasmid)	100	100	Yes
AAQ17158	sensor histidine kinase VanS (plasmid)	100	100	Yes
AAQ17161	vancomycin B-type resistance protein VanX (plasmid)	100	100	Yes
AAL07292	D,D-dipeptidase VanXb, partial	99.44	99.44	Yes
AAQ17162	D-alanyl-D-alanine carboxypeptidase VanY (plasmid)	99.66	100	Yes
AAQ17163	vanZ protein (plasmid)	100	100	Yes
EUJ19660	hypothetical protein MAQA_05683	37.50	53.33	No
EUJ18463	2-C-methyl-D-erythritol 2,4-cyclodiphosphate synthase	46.67	47.06	No
EUJ21751	lysyl-tRNA ligase	41.18	46.51	No
EUJ18464	glutamyl-tRNA ligase	53.33	40.91	No
EUJ17220	30S ribosomal protein S21	53.85	56	No
CDC69830	purine-nucleoside phosphorylase	41.18	40.83	No
CDC71755	putative uncharacterized protein	63.64	39.53	No
CDC68822	dNA gyrase subunit A	40.74	36.67	No
CDC68565	primosome assembly protein PriA	52.94	39.62	No
WP_018370009	HU family DNA-binding protein	37.25	62.96	No

WP_018370157	serine O-acetyltransferase	50.00	55.32	No
WP_018369970	DNA polymerase III subunit delta′	41.67	42.86	No
WP_018369925	segregation/condensation protein B	34.38	46.15	No
WP_018370631	chromosome segregation protein SMC	38.46	39.66	No

Unique protein names with NCBI accession numbers obtained from *Clostridium* and *Enterococcus*

Table S3: List of 25 sequences that convey resistance to *bac*.

NCBI accession number	Protein name
AAK78481	Bacitracin resistance protein (bacA)
AAK78939	Bacitracin resistance protein
AAO35165	bacitracin resistance protein
ABG83114	undecaprenyl-diphosphatase UppP
ABR33181	putative undecaprenol kinase
ABS32525	undecaprenyl-diphosphatase UppP
ABS34716	undecaprenyl-diphosphatase UppP
ACD23687	undecaprenyl-diphosphatase UppP
BAH07085	hypothetical protein CKR_2034
BAH07444	hypothetical protein CKR_2393
EDS74459	undecaprenyl-diphosphatase UppP
EDS77743	putative undecaprenol kinase
EDT76304	putative undecaprenol kinase
EDT76517	putative undecaprenol kinase
EDX92686	undecaprenol kinase
EEA85136	undecaprenyl-diphosphatase UppP
EEH97446	undecaprenyl-diphosphatase UppP
ZP_01802436	hypothetical protein CdifQ_04003416
ZP_01804067	hypothetical protein CdifQ_04001473
ZP_02211008	hypothetical protein CLOBAR_00606
ZP_02427934	hypothetical protein CLORAM_01322

ZP_03291854	hypothetical protein CLONEX_04087
AAS78449	BcrD (plasmid)
EEI10697	undecaprenyl-diphosphatase UppP
EEI12255	undecaprenyl-diphosphatase UppP

Table S4: List of 52 sequences that convey resistance to *van*.

**NCBI accession number**	**Protein name**
AAA65956	vanA
ACL82961	VanM
EEI12987	D-ala D-ala ligase N-terminal domain protein
AAA24786	D-alanine-D-alanine ligase-related protein
AAD41882	D-alanine:D-lactate ligase
AAG37026	D-alanine:D-lactate ligase
AAZ98839	VanD
AAL27442	VanE
AAF71281	ligase
ABB17543	VanG2
AAA24789	vancomycin resistance protein
AAV58815	vanHB
AAD41881	D-specific alpha-keto acid dehydrogenase
AAG37025	alpha keto acid dehydrogenase
AAA24787	vancomycin response regulator
AAB05622	response regulator
AAF86641	response regulator VanRc
AAK53981	VanRc-2
AAD42180	response regulator
AAL27445	VanRE
AAQ16268	response regulator
ABB17540	VanRG2
AAA24788	vancomycin histidine protein kinase

AAV58812	vanSB
AAF86642	sensor-histidine kinase VanSc
ABX79412	VanSc4
AAD42181	protein kinase
AAZ98836	VanSD
AAL27446	VanSE
AAQ16269	histidine protein kinase
AAD22403	serine racemase VanT
AAL27444	VanTE
AAQ16274	serine racemase
AAQ16267	predicted transcriptional regulator
EEI12985	VanW-like protein
AAF71280	VanWG
AAQ16271	VanWG
AAA65957	vanX
AAV58817	vanXB
AAD42185	D,D dipeptidase
AAM09852	D,D-dipeptidase
AAZ98840	VanXD
AAF61331	D,D-dipeptidase/D,D-carboxypeptidase VanXYc
AAK53979	VanXYc-2
AAL27443	VanXYE
AAF71282	D,D-peptidase
AAA65958	vanY
AAV58813	vanYX
AAD42182	D,D carboxypeptidase
ACM47285	D,D-carboxypeptidase VanYD
ABA71729	VanYG
AAA65959	teicoplanin resistance protein

Table S5: List of 52 essential gene sequences that do not convey resistance to *bac* or *van*.

**NCBI accession number**	**Protein name**
WP_048604623	MULTISPECIES: chromosomal replication initiator protein DnaA
WP_016251883	diacylglycerol kinase family protein
WP_048605016	sodium:proton antiporter
WP_048604147	type I 3-dehydroquinate dehydratase
ZP_05713806	shikimate kinase
WP_048604156	divalent metal cation transporter
WP_048605120	MULTISPECIES: primosomal protein DnaI
WP_048603139	segregation/condensation protein A
WP_019723580	DNA primase
WP_065096579	CCA tRNA nucleotidyltransferase
WP_048603010	transcription termination/antitermination protein NusA
WP_048604989	hypothetical protein
WP_047242264	o-succinylbenzoate synthase
WP_147570559	SMC-Scp complex subunit ScpB
WP_048604535	chromosome segregation protein SMC
WP_047242017	magnesium transporter CorA family protein
WP_047241929	primosomal protein DnaI
WP_010709765	MULTISPECIES: hypothetical protein
WP_016251515	NUDIX hydrolase
WP_016252455	HU family DNA-binding protein
ZP_05713234	amidohydrolase
WP_060470251	alpha/beta hydrolase
WP_047340880	chromosome segregation protein SMC
WP_060470869	DNA primase
WP_029594152	SulP family inorganic anion transporter
OJG33691	hypothetical protein RT42_GL001961
CDA16793	dephospho-CoA kinase
CDA16801	thioredoxin-disulfide reductase

CDA16620	50S ribosomal protein L3
CDA15955	nusA antitermination factor
CDA16279	l-lactate dehydrogenase 2
CDA16644	30S ribosomal protein S11
CDA16929	alanine racemase
CDA16646	dNA-directed RNA polymerase subunit alpha
CDA16519	cTP synthase
CDA16766	putative ABC transporter permease protein
CDA15715	30S ribosomal protein S12
CDA16670	bifunctional protein FolD
CDA16076	putative 4Fe-4S binding domain protein
CDA16363	dNA-directed RNA polymerase subunit beta
CDA16869	4-hydroxy-3-methylbut-2-en-1-yl diphosphate synthase
CDA16969	50S ribosomal protein L20
CDA16154	dNA protection during starvation protein 2
CDA16765	o-antigen export system ATP-binding protein RfbB
CDA15652	putative uncharacterized protein
CDA16736	formate–tetrahydrofolate ligase
CDA16647	50S ribosomal protein L17
CDA16211	lysine–tRNA ligase
CDA16624	30S ribosomal protein S19
CDA16718	primosomal protein N′
CDA16814	anaerobic ribonucleoside-triphosphate reductase
CDA16591	aspartate carbamoyltransferase
